# Atypical presentation of cerebellar posterior reversible encephalopathy syndrome in a patient with HIV

**DOI:** 10.1002/ccr3.2625

**Published:** 2020-01-11

**Authors:** Michael Raya, Imaad Nasir, Antonio Liu

**Affiliations:** ^1^ Department of Neurology Adventist Health White Memorial Los Angeles California; ^2^ Department of Neurology University of Arizona Banner University Medical Center Tuscson Arizona

**Keywords:** atypical posterior reversible encephalopathy syndrome, PRES HIV

## Abstract

This case report is meant to widen the scope of lesion locations in posterior reversible encephalopathy syndrome (PRES) to include the brainstem, frontal lobe, and basal ganglia in order to increase the diagnosis and treatment of PRES.

## Graphical abstract

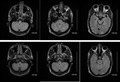

## 1 BACKGROUND

We present here a patient with HIV, end stage renal failure on hemodialysis, and uncontrolled essential hypertension who reported headaches and blurred vision on admission; he was found to have posterior reversible encephalopathy syndrome (PRES) in the cerebellum. The finding of PRES in an atypical location helps aid in establishing a more robust criteria for diagnosis and timely treatment of PRES.

Posterior reversible encephalopathy syndrome is a clinically frightening and easily misdiagnosed condition. Clinical presentations include seizures, encephalopathy, headaches, visual disturbances, or other focal neurological deficits.^1^, ^2^ Clinicians rely on neuroimaging and the sudden appearance of neurological deficits to make their diagnosis. However, this clinical alarm is not always reliable as increased T2 and fluid attenuated inversion recovery signal in the posterior regions of the cerebral hemisphere are not always present on imaging.^3^ This report highlights an instance of PRES with atypical cerebellar lesions. Increased awareness of atypical presentations of PRES will aid clinicians in making a timely diagnosis and preventing a potentially life‐threatening complication, such as ischemia or intracranial hemorrhage. PRES in association with atypical locations has been reported before,^4^, ^5^ however, additional reports are needed until this exception to the rule becomes part of the broader understanding of PRES.

## 2 CASE PRESENTATION

A 29‐year‐old man with HIV, end stage renal disease on hemodialysis, and hypertension presented to the Emergency Department with a 2‐day history of headache and blurry vision. He described his headache as generalized and nonthrobbing. The patient reported compliance with all his medications including antiretroviral therapy and antihypertensive medications. He denied history of smoking, drinking, or drug use.

On physical examination, the patient was noted to be afebrile and saturating well in room air with a blood pressure of 245/141. He was alert and oriented, and visual acuity is 20/80 in both eyes. There was no scotoma or visual field deficits; neck was supple, CN II‐XII intact, reflexes +2, strength 5/5, and sensation intact in all four extremities. No dysarthria, aphasia, dysphagia, dysmetria, or dysdiadochokinesis was noted, and the rest of examination was unremarkable. CBC was within normal limits. The patient had a CD 4 count of 325 and HIV‐1 RNA Qnt RT PCR of 5100. CT head obtained in ED was negative, and subsequent lumbar puncture with CSF analysis showed normal opening pressure, clear fluid with WBC count of 2 and protein count of 39. CSF Indian Ink, cocci, crypto, HSV, and VDRL were all negative. Urine toxicology screen was also negative.

**Figure 1:**
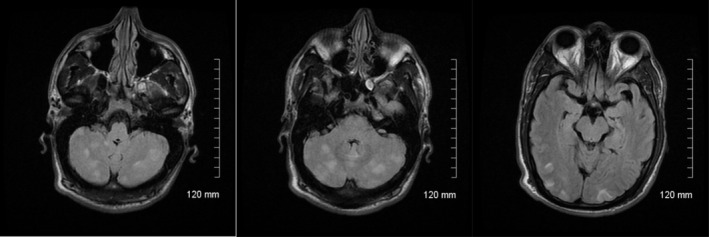
MRI FLAIR sequence on day 2

MRI on day 2 of hospitalization (Figure 1) showed no DWI or ADC mapping abnormality, but there were definite abnormal signal densities in different sequences suggesting PRES. In addition to the typical posterior area, these characteristic changes were also present in the cerebellum.

**Figure 2:**
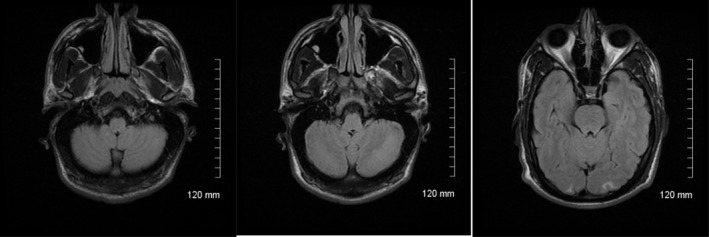
MRI FLAIR sequence on day 5

Throughout his hospital stay, he received aggressive blood pressure management and his symptoms gradually subsided. By day 5, his blood pressure is 147/86 and a repeat MRI clearly showed resolution of most lesions. The cerebellar lesions completely resolved (Figure 2).

Patient was discharged after day 7 of hospitalization completely asymptomatic. He was called on the phone three months after discharge and stated that his symptoms have continued to be completely resolved.

## 3 DISCUSSION

Our patient is a 29‐year‐old man with HIV, end stage renal disease on hemodialysis and hypertension who presented with a two‐day history of headache, blurry vision, along with a blood pressure of 245/141. Concern for ischemic stroke, cerebral hemorrhage, cerebral vein thrombosis, and hypertensive emergency was among the immediate differential diagnoses. Subsequent CT investigation and fundoscopic examination were normal. One of the main proposed mechanisms of PRES is the failure of cerebral autoregulation when blood pressure exceeds the upper limit of normal. In the acute setting, hypertension above the upper limit of cerebral autoregulation leads to arteriole dilation of the brain causing the breakdown of the blood‐brain barrier and endothelial dysfunction in regions of the brain. It is theorized that the sympathetic innervation of the arterioles helps persevere the blood‐brain barrier from marked increased blood pressures and is noted to be higher in concentration in the anterior circulation than the posterior circulation of the brain.^1^, ^6^ Additional causes of endothelial dysfunction reported in cases of PRES include renal disease, preeclampsia/eclampsia, autoimmune diseases, AIDS, TTP, sepsis, and immunosuppressive agents.

The patient's physical examination revealed no focal neurological deficits, and his laboratory workup excluded an infectious or toxic etiology. Despite a clinical scenario strongly suggesting PRES, a true distinction cannot be made until MRI findings are observed, and those positive findings are reversed, as the name of the syndrome implies.^7^ Day 2 MRI findings confirmed lesions in the parieto‐occipital region, which is consistent with the most common trend of patients with established diagnosis of PRES. The additional cerebellar lesions seen in this patient are less commonly reported, but this may be attributed to misdiagnosis by providers who have a lower index of suspicion for nonposterior white matter lesions in the clinical setting of PRES. With aggressive antihypertensive therapy, the patient improved clinically, and lesions were resolved on MRI by day 5.

This case report argues that widening the scope of location of lesions in PRES to include the brainstem, frontal lobe, and basal ganglia may allow for increased diagnosis of PRES in cases where the posterior area is not affected, or not affected exclusively. While PRES continues to be prominent for the “posterior” location of the lesions, failing to recognize PRES in an atypical location on neuroimaging could result in catastrophic consequences including irreversible cytotoxic edema.^8^ Additionally, the exact mechanism of PRES remains controversial and unproven, as cases of vasospasm, hypoperfusion, as well as hyperperfusion have all been documented.^1^, ^4^ Cases such as ours will aid in establishing a more robust criteria for diagnosis and timely treatment of PRES.

## CONFLICT OF INTEREST

None declared.

## AUTHOR CONTRIBUTIONS

Michael Raya, MD—Department of Neurology, Adventist Health White Memorial, CA.—has been involved in revising manuscript for important intellectual content. Imaad Nasir, MD—Department of Neurology, University of Arizona, Banner University Medical Center, AZ.—has made substantial contributions to acquisition of data and has been involved in drafting the manuscript. Antonio Liu, MD—Department of Neurology, Adventist Health White Memorial, CA.—involved in overseeing attending physician and given final approval of the version to be published.
